# Healthy ageing trajectories and lifestyle behaviour: the Mexican Health and Aging Study

**DOI:** 10.1038/s41598-019-47238-w

**Published:** 2019-07-30

**Authors:** Christina Daskalopoulou, Artemis Koukounari, Yu-Tzu Wu, Graciela Muniz Terrera, Francisco Félix Caballero, Javier de la Fuente, Stefanos Tyrovolas, Demosthenes B. Panagiotakos, Martin Prince, Matthew Prina

**Affiliations:** 1 Department of Health Service and Population Research, King’s College London, Institute of Psychiatry, Psychology and Neuroscience, London, UK; 2 Department of Infectious Disease Epidemiology, London School of Hygiene & Tropical Medicine, Faculty of Epidemiology and Population Health, London, UK; 3 Centre for Dementia Prevention, Centre for Clinical Brain Sciences, University of Edinburgh, Edinburgh, UK; 4 Department of Preventive Medicine and Public Health and Microbiology, Universidad Autónoma de Madrid, Madrid, Spain; 5 Ciber of Epidemiology and Public Health, Carlos III Institute of Health, Madrid, Spain; 6 Hospital Universitario de La Princesa, Instituto de Investigación Sanitaria Princesa (IIS Princesa), Madrid, Spain; 7 Department of Psychiatry, Universidad Autónoma de Madrid, Madrid, Spain; 8 Parc Sanitari Sant Joan de Déu, Universitat de Barcelona. Fundació Sant Joan de Déu, Dr Antoni Pujades, 42, 08830, Sant Boi de Llobregat, Barcelona, Spain; 9 Instituto de Salud Carlos III, Centro de Investigación Biomédica en Red de Salud Mental, CIBERSAM, Madrid, Spain; 10 Department of Nutrition and Dietetics, School of Health Science and Education, Harokopio University, Athens, Greece

**Keywords:** Risk factors, Public health

## Abstract

Projections show that the number of people above 60 years old will triple by 2050 in Mexico. Nevertheless, ageing is characterised by great variability in the health status. In this study, we aimed to identify trajectories of health and their associations with lifestyle factors in a national representative cohort study of older Mexicans. We used secondary data of 14,143 adults from the Mexican Health and Aging Study (MHAS). A metric of health, based on the conceptual framework of functional ability, was mapped onto four waves (2001, 2003, 2012, 2015) and created by applying Bayesian multilevel Item Response Theory (IRT). Conditional Growth Mixture Modelling (GMM) was used to identify latent classes of individuals with similar trajectories and examine the impact of physical activity, smoking and alcohol on those. Conditional on sociodemographic and lifestyle behaviour four latent classes were suggested: high-stable, moderate-stable, low-stable and decliners. Participants who did not engage in physical activity, were current or previous smokers and did not consume alcohol at baseline were more likely to be in the trajectory with the highest deterioration (i.e. decliners). This study confirms ageing heterogeneity and the positive influence of a healthy lifestyle. These results provide the ground for new policies.

## Introduction

The number of people 60 years old and over is increasing and many parts of the world will experience a significant growth in the next decades^1^. An increased life expectancy is the result of medical and technological advances together with better social and environmental conditions. However, living longer does not entail that these added years will be spent in good health as there is contradictory evidence that older people nowadays age with better health compared to their parents^2,3^. Furthermore, population ageing has been associated with an increased risk of non-communicable diseases^4^, disability^5^ and frailty^6^. All these put extra challenges on the already stretched public health and social care sectors.

Reviews indicated that many studies have examined various factors which influence the health of older people in a positive or a negative way^7,8^. However, the vast majority of research has assumed that a single ageing profile, in which good health is followed by rapid decline and then death, is representative of all older people. Nevertheless, recent findings suggest that ageing is a heterogeneous process and that no typical ageing profile exists^9^. To be able to provide valuable insight to policymakers and clinicians on the various ageing profiles, there is a need to identify those factors that have the largest effect on the interindividual heterogeneity of getting older. Furthermore, identifying the interrelationships of risk factors with the various ageing pathways could contribute to more targeted strategies, and hence more effective, that could enable people to prevent or control any negative health outcomes.

Even though population ageing is a global phenomenon, areas of the world have been experiencing a different demographic transition. Latin American countries and the Caribbean figure among those that are projected to experience the fastest population growth in the following decades^1^. Population ageing in Mexico has been characterised as America’s big challenge as the proportion of people above 65 years old is projected to triple by 2050^10^. In addition, in Mexico the epidemiological transition from communicable diseases to non-communicable diseases has been linked with an unprecedented increased risk of non-chronic diseases (i.e. diabetes, chronic kidney disease)^11^. However, ageing research in Mexico is very limited; especially, with regards to the different ageing profiles and their associations with protective or risk factors^8^. Recent systematic reviews highlight the positive effect of modifiable lifestyle factors, in particular physical activity and smoking abstinence, on healthy ageing^12,13^. Yet, in Mexico smoking continues to be a serious public health problem and one of the most important risk factors of diseases and mortality^14^. In addition the proportion of people being physically inactive, especially among older Mexicans, has been increased during the last years^15^. An increase in the detrimental use of alcohol consumption, mainly binge drinking, has also been observed lately^16^.

The purpose of this study was to identify subgroups of older Mexicans exhibiting similar health trajectories over the later years of the life course and to examine the effect of physical activity, non-smoking and alcohol consumption on those across 14 years of follow-up. In our study, health in older people was conceptualised within the functional ability framework as suggested by the latest report of Health and Ageing from the World Health Organisation (WHO). More specifically, WHO defined healthy ageing as “the process of developing and maintaining the functional ability that enables older people to do the things that matter to them”^17^. Functional ability is comprised by the intrinsic capacity of an individual, physical and mental capacities, and the surrounding environment (i.e. community, home, devices). Within this framework, more focus is based on function than the presence of any disease or comorbidity^18^.

## Design and Methods

### Study sample

The Mexican Health and Aging Study (MHAS) is the first urban-rural nationally representative longitudinal study of older adults in Mexico. The main goal of the MHAS was to examine the ageing process and the disease and disability burden of people 50 years old and over from various socioeconomic backgrounds^19,20^. The study protocol and instruments were approved by the Institutional Review Board or Ethics Committee of the University of Texas Medical Branch, the INEGI and the Instituto Nacional de Salud Pública in Mexico. Freely accessible datasets and detailed documentation are provided (www.MHASweb.org).

The baseline survey took place in 2001 and there are three follow-up waves available; 2003, 2012 and 2015. Data were obtained from face-to-face interviews and in case where the participant was absent or in poor health, proxy interviews took place. For the needs of the current study, we considered data from direct interviews of participants who were firstly interviewed in 2001 and then followed-up. We did not include participants who were firstly interviewed in a follow-up wave.

### Indicators of health

In this study, to conceptualise health in older age, we adopted the functional ability framework as provided by WHO^17^. Items of functional ability and measured tests were identified in the 4 waves to create a metric of health status in old age. A set of 40 items providing information on difficulties of activities of daily living (ADLs) and on instrumental activities of daily living (iADLs), together with items measuring pain, sleep and energy problems, and cognitive tests were identified in the 4 waves. 30 items were available in all waves and constitute the anchor items (i.e. items contributing to parameters linkage) (Supplementary Table S1). To create the health metric, we included items available in all waves and items measured in at least 2 waves.

Answers to the items were recoded to define presence or absence of the difficulty, items with adverse coding were recoded accordingly. Participants who refused or declined to answer a question were handled as missing cases (less than 1% on average in the 4 waves); responses from participants who answered “cannot do the activity” were recoded as “having the difficulty” whereas responses from participants who answered “do not do the activity” were recorded as “not having the difficulty”. Hearing and eyesight condition were recoded as good when participants replied excellent, very good, good or fair and poor when they replied poor or worse. In our study verbal fluency was examined by the number of animals remembered in one minute; participants with values ≤ 25^th^ percentile were characterised as having low ability.

### Covariates

In our models we considered age, sex, educational level, physical activity, smoking and alcohol consumption as covariates. Educational level was grouped as ‘none, primary, secondary, technical or commercial, preparatory or high school, basic teaching school, college, and graduate’ with higher values indicating higher level. Physical activity was captured via a single question asking participants if on average during the last 2 years they had exercised or done hard physical work 3 or more times a week, including various activities such as sports, heavy household chores, or other physical work. Smoking history was assessed by a single question asking if the individual had smoked more than 100 cigarettes or 5 packs in his lifetime. Alcohol history was assessed in a similar way by asking participants if ever drink alcoholic beverages.

## Analytical Procedure

### Health metric

To fully capture the underlying latent construct of health in older age, we created a measurement model. The measurement model includes parameters that represent the difficulty and the discriminatory power of each question/ item. By this approach, items are allowed to differ in their relative difficulty and discrimination ability. Thus, we are able to differentiate participants with similar levels of health. More specifically, to create a common metric of health we employed Bayesian multilevel Item Response Theory (IRT) and estimated a two parameter normal-ogive model^21^. Item parameters (difficulty and discrimination) determine the exact relationship between the latent trait and the probability of the response to a particular item^22^. The IRT model assumes a one-dimensional continuous latent variable (in our study the health trait) that predicts the probability of a certain observed response to each item.

A random item effects (multilevel) approach was implemented to take into account the multilevel structure of the data and allow item parameters to vary across waves. By this approach wave-specific parameters are assumed to follow a normal distribution: b_ij_~N(b_i_, σ_b,i_^2^), a_ij_~N(a_i_, σ_a,i_^2^) (b: difficulty; a: discrimination; σ: standard deviation; i: item; j: wave) and to be random deviations from the overall item “hyperparameters”^23^. In a multilevel IRT model, a “hierarchical prior distribution or hyperprior” can also be assumed for the hyperparameters: b_i_~N(μ_σ_, ω_b_^2^); a_i_~N(1, ω_a_^2^). The Bayesian framework was adopted as it allows to simultaneously estimate all parameters under a Markov Chain Monte Carlo (MCMC) estimation method and to also include different sets of items per wave^24^.

We compared 4 potential models according to the estimated variance components in the item parameters across the 4 waves: (1) no variance in the difficulty and discrimination parameters (σ_b,i_^2^ and σ_a,i_^2^ were assumed to be zero); (2) item-specific difficulty variance and no variance in the discrimination parameters (σ_b,i_^2^ is estimated); (3) homogeneous difficulty variance and no discrimination variance (a joint variance of all item difficulties is estimated σ_b,1_^2^ = σ_b,2_^2^ = … = σ_b,I_^2^); (4) variance in difficulty and discrimination parameters and estimation of the hyperprior distribution (σ_b,i_^2^ and σ_a,i_^2^ are estimated and hyperpriors with parameters μ_σ_, ω_b_, ω_a_). In models (1), (2) and (3) the discrimination parameters (a_ij_) were fixed at one. For identification purposes, in all models and for each wave the sum of all difficulty parameters (b_i_) was fixed to zero and the product of all discrimination parameters (a_i_) was fixed to one^25^. More information regarding the technical settings of the model and the priors of the parameters are available in the *sirt* package documentation^26,27^ in R 3.5.1 statistical software^28^. 7,000 samples were used for parameter estimation and the first 100 samples were discarded (burn-out).

To identify the model that provided the best fit in our data we examined the Expected-A-Posteriori (EAP) estimation reliability, the Deviance information criterion (DIC), the precision of the measurement and the R-hat MCMC convergence statistic. Higher values in EAP reliability indicate a higher reliability of the metric whereas lower values in DIC indicate a model which is better supported by the data^29^. The measurement precision is considered appropriate when the standard errors (SE) are below 0.5 for most of the spectrum of the latent construct^30^. Finally, R-hat values substantially above 1 indicate lack of convergence for the MCMC algorithm^31^. The final extracted health metric score was transformed in a scale 0–100 with higher scores indicating better health.

IRT models assume unidimensionality of the latent construct^22^. We investigated this assumption by performing exploratory factor analysis (EFA) on a sub-sample of the initial baseline sample (70%) under a goemin (oblique) rotation. A second-order confirmatory factor analysis (CFA) was subsequently performed on the validation sub-sample (30%) to confirm or not that health could be represented as a single general construct. Analyses were performed in Mplus v8.0^32^ with the mean and variance-adjusted weighted least-squares (WLSMV) estimator and a pairwise present approach to missing data^33^. To conclude about the goodness-of-fit of the models, we examined the comparative fit index (CFI) and the root mean square of approximation (RMSEA) with 90% confidence intervals (CI). We considered a model to have an acceptable fit when CFI ≥ 0.90 and RMSEA values close or less than 0.06^34^.

Finally, to confirm the predictive validity of the health metric, we performed a Receiver Operating Characteristics (ROC) curve analysis adjusted per gender. Mortality was assumed as the gold-standard measure and we examined the associations of the baseline metric (2001) with mortality observed over increasing periods of time such as: 2 years (2003), 11 years (2012) and 14 years (2015) by calculating the Area Under the ROC Curve (AUC). ROC analyses were implemented in STATA^35^.

### Trajectories of health

We used growth mixture modelling (GMM) to investigate the longitudinal trajectory of unobserved groups (latent classes) with similar patterns of health in older age^36^. By this approach, we can identify ‘mixtures” of two or more homogeneous subpopulations in the total population^37^. GMM provides information regarding the optimal number of classes, the number of people in each class, predictors of class membership as well as the growth factors of each different trajectory. Growth factors usually entail the intercept and the slope; the level of outcome variable when time is equal to zero and the rate of change in the outcome over time, respectively (interpretation is also dependable on the way the model has been parameterised).

In agreement with current recommendations^36^, we initially performed a single-group analysis to determine the pattern of change over time. The number of available time points (i.e. 4 waves) allowed us to examine a linear, a latent basis and a quadratic pattern of change^38^. We then applied a conditional GMM (i.e. we included the covariates as previously described) with a distal outcome approach to identify latent classes in terms of their health trajectories within our dataset^39^. We employed an exploratory approach and we fitted models with an increasing number of classes to identify the optimal latent class model. We also investigated several sets of models in which we allowed for the mean, variances and/or covariance of the intercepts and slopes to differ among latent classes. Missing data were assumed to be missing at random (MAR) and listwise deletion was applied to cases that had missing values on covariates.

To estimate the number of latent classes, we followed recommended approaches including the comparison of various model fit statistics, substantive meaning and interpretability of each class^39^. We inspected the Bayesian information criterion (BIC), the sample-size adjusted BIC (SSABIC), entropy values and the Lo-Mendel-Rubin likelihood ratio test (LMR-LRT)^40^. Lower BIC and SSABIC values indicate a more parsimonious and better fitting model, whereas higher entropy values signal better class separation^41^. Sample size of the smallest class was also considered^42^. Models were estimated in Mplus v8.0 by full maximum likelihood (FML) and robust standard errors (MLR) to non-normality and non-independence of observations^32^. To avoid local maxima for the EM (expectation-maximization) algorithm, we estimated the models with 250 random starting values.

**Figure 1:**
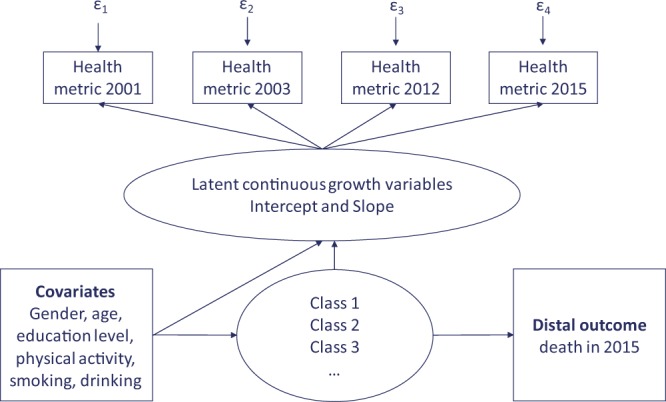
General diagram of the conditional growth mixture model used in the study. Squares represent observed variables; circles represent latent (unobserved) variables or factors; ε (epsilon) represents the measurement error; classes represent the various unobserved groups of individuals with similar patterns of health; the distal outcome -observed mortality status in 2015- indicates the predictive value of the health metric.

It has to be pointed out that we applied a conditional GMM and investigated how classes are influenced and predicted by the covariates. We opted for a one-step approach examining the association between latent class variable and covariates to avoid estimation errors occurring when participants are forced to be classified in one-single class^43^. Age, sex, education level, physical activity, smoking and alcohol consumption were simultaneously included as covariates on the intercept and slope, and as markers of class membership. By this way, the different associations of each covariate with the latent classes was assessed, controlling for all other covariates. Since the latent classes are categorical, the estimated associations are from a multinomial logistic regression. Consequently, the estimates represent the log odds of being in a non-reference latent class versus being in the reference. We also considered mortality status in 2015 (dead or alive) as a distal outcome of the latent classes to more clearly indicate the predictive value of the trajectories^44^. The model implemented is depicted in Fig. 1.

## Results

Table 1 provides baseline descriptive statistics of the participants. Our sample comprised 14,143 individuals in the baseline (5,920 men, 8,195 women) with a mean age of 59.99 (SD:10.66); the majority had at least above primary level education (76.4%). Almost one-third of them (33.7%) reported that they had done some physical activity within the last 2 years more than 3 times per week and that they drink alcohol beverages (31.3%). Individuals were fairly divided in ever smokers (42.7%) and non-smokers (57.3%). Missing values on the covariates were trivial (<0.8%).

**Table 1:** Descriptive statistics for the baseline wave (2001).

Variables	Baseline	14,143
Age	Mean-SD	59.99 (10.66)
	Missing	28 (0.2%)
Sex	Males	5,920 (41.9%)
	Females	8,195 (57.9%)
	Missing	28 (0.2%)
Education Level	None	3,325 (23.5%)
	Primary	7,527 (53.2%)
	Above secondary	3,282 (23.2%)
	Missing	9 (0.1%)
Physical Activity	Yes	4,765 (33.7%)
	No	9,264 (65.5%)
	Missing	114 (0.8%)
Ever smoked	Yes	6,041 (42.7%)
	No	8,099 (57.3%)
	Missing	3 (0.02%)
Drinking alcohol	Yes	4,420 (31.3%)
	No	8,332 (58.9%)
	Never has used alcohol	1,385 (9.8%)
	Missing	6 (0.04%)

Notes: SD: standard deviation.

### Health metric

**Figure 2:**
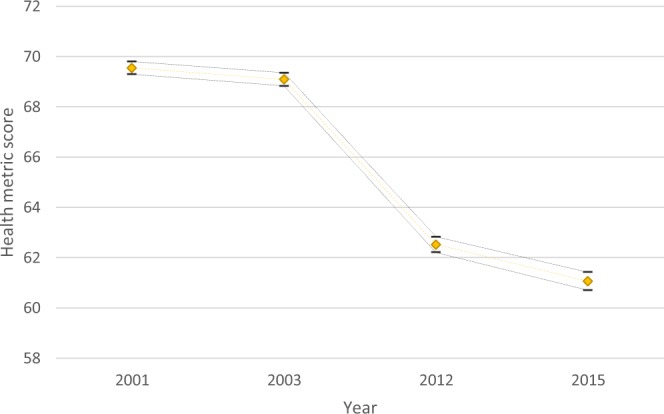
Health metric scores per measurement year (2001, 2003, 2012, 2015). Diamond markers represent mean values of the health metric score; dash markers represent the upper and lower bound of the 95% confidence interval for the mean value.

To estimate health in older age, model fit diagnostics concluded that the best fit model in our dataset was Model 3, which allowed a homogeneous variance across waves for the difficulty parameter to be estimated (Supplementary Tables S2–S6). According to the difficulty parameters of the IRT model the most difficult items were those referring to mental abilities (i.e. visual recall, learning ability) and the least difficult were items of iADL (i.e. difficulty eating or taking medications due to health problem). The EFA results indicated that a four-factors model was the best solution to the latent structure of our dataset (χ^2^: 4,985.58, df: 402, RMSEA: 0.034; 90%CI: 0.033–0.035, CFI: 0.977); however intercorrelations among the first-order factors provided support for a higher-order factor (Supplementary Table S7). The second-order CFA in the 30% sub-sample confirmed that a general factor, comprised by the initial four factors of the EFA, underlies the data (χ^2^: 3,413.5, df: 491, RMSEA: 0.037; 90%CI: 0.036–0.039, CFI: 0.964) providing enough evidence for unidimensionality. Regarding mortality, 29% (n = 4,033) of the baseline sample was dead by 2015, 65% (n = 9,223) were found alive and no information was available for 6% (n = 887). The gender-adjusted AUC associated with the baseline (2001) health metric for the 2003, 2012 and 2015 mortality assessments was: AUC: 0.75 (95%CI: 0.73–0.78); AUC: 0.71 (95%CI: 0.69–0.72); AUC: 0.70 (95%CI: 0.69–0.71), respectively. The health score indicated a decreasing trend across the four waves Fig. 2.

### Trajectories of health

To ensure that we identified the model of change that best represented the four waves of data, we conducted three single-group analyses. These showed that the latent basis model was the most appropriate to model the shape of change over time (lowest BIC/ SSABIC values) (Supplementary Table S8). A final sample of 13,988 participants (out of the initial 14,143) was included in our conditional GMM analyses due to missing data on covariates. The lowest covariance coverage for each pair of variables was 0.45 (obtained using Mplus). Hence the missing values were within acceptable limits for the analyses (minimum threshold for model convergence is 0.10). As noted in our analytical procedure, we examined various sets of models regarding the means, variances and covariances of the growth factors across the latent classes. However, due to identification and convergence reasons, we proceeded by assuming equal intercept and slope variances among latent classes.

Table 2 provides the BIC, SSABIC, entropy values and the adjusted LRT results for the one-, two-, three-, four- and five-classes models. The four-class model was selected according to the BIC/SSABIC indexes and in combination with the entropy and the adjusted LRT. This model had the lowest BIC and even thought the adjusted LRT and SSABIC suggested as best the five latent class model, the considerable drop of the entropy (0.58) and the low-size classes (<5% of the total sample) indicated as better the former. Between the three and four-class models, we proceeded with the latter as LRT indicated.

**Table 2:** Model Selection Criteria of the Growth Mixture Model (GMM) analysis.

Fit Statistics	2 Classes	3 Classes	4 Classes	5 Classes	6 Classes
LL (N)	−166,428.21 (46)	−166,037.18 (68)	−165,836.24 (90)	−165,739.89 (112)	n/a
BIC	333,295.53	332,723.48	**332**,**531**.**61**	332,548.93	
SSABIC	333,149.34	332,507.38	332,245.60	**332**,**193**.**00**	
Entropy	0.746	**0**.**760**	0.710	0.578	
Adj. LMR-LRT	−168,423.35^*^	−166,428.21^*^	−166,037.18^*^	−165,836.24^*^	
Group size (%) C1	26.9%	68.9%	22.6%	32.9%	
C2	73.1%	23.7%	13.0%	4.7%	
C3		7.4%	59.0%	10.7%	
C4			5.4%	20.9%	
C5				30.9%	

Notes: LL: Log Likelihood; N: number of parameters; BIC: Bayesian Information Criterion; SSABIC: Sample size adjusted Bayesian Information Criterion, Adj. LMR-LRT: adjusted likelihood ratio test; n/a: no convergence; *p-value < 0.05.

**Figure 3:**
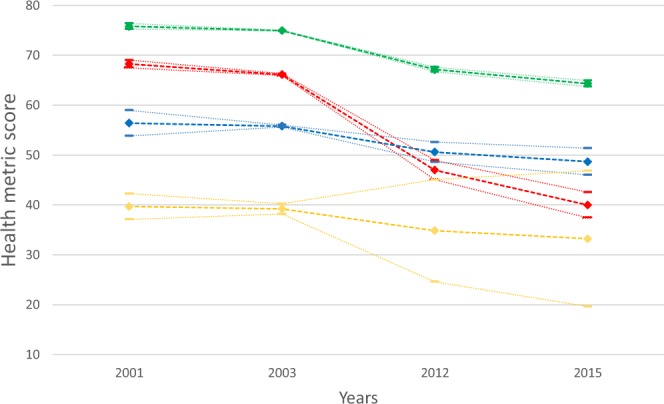
Trajectories of the conditional 4-class model. Diamond markers represent the estimated mean health score per group across the four measurement waves (years 2001, 2003, 2012, 2015). Dash markers represent the upper and lower bound of the 95% confidence interval for the mean values. Red points represent the decliners group (n = 3.161); yellow points represent the low-stable group (n = 756); blue points represent the moderate-stable group (n = 1.824) and green points represent the high-stable group (n = 8.247).

Figure 3 provides the trajectories for the latent classes in the four-classes conditional model. Based on the growth factors, the first class (red) was named “decliners” group. There were 3,161 individuals (22.6% of the sample) with an average baseline health score of 68.27 (SE:0.40) and a steep average decline rate of −28.29 (SE:1.31) in the follow-up waves. The second class (blue) named “moderate-stable” had 1,824 individuals (13.0%) with moderate level of health in baseline (intercept:56.39, SE:1.31) and moderate decline (slope:-7.71, SE:1.36). The largest class (class 3-green) called “high-stable” had 8,247 participants (59% of the sample). In this group, there were those with high average baseline health score (intercept:75.83, SE:0.29) and moderate average rate of decline -11.52 (SE:0.31). The smallest class (class 4-yellow) named “low-stable” had 756 participants who demonstrated low average baseline scores (intercept:39.69, SE:1.32) and no significant average rate of change (slope:-6.46, SE:6.94) in the follow-up waves. Decliners and low-stable groups showed the highest death probability in 2015; 0.81 (SE:0.02) and 0.95 (SE:0.03), respectively. The moderate-stable group showed a moderate death probability after 14 years of follow-up 0.26 (SE:0.04), whereas the high-stable had the smallest death probability 0.05 (SE:0.01).

### Covariates

#### Lifestyle behaviour as predictors of class membership

Table 3 shows the logit coefficients as well as the odds ratios from the multinomial logistic regression of the latent classes on the lifestyle behaviour factors adjusted for socio-demographics. With high-stable group as the reference class, membership in the decliners and in the low-stable group was associated with physical activity, smoking and alcohol consumption. Non-physically active participants had greater odds of being in the decliners group (OR:1.39; 95%CI:1.17–1.66) and even greater (OR:8.76; 95%CI:3.92–19.56) in the low-stable group compared to the high-stable group. On the contrary, non-smokers had decreased odds of being in the decliners (OR:0.68; 95%CI:0.57–0.81) or in the low-stable group (OR:0.56; 95%CI:0.38–0.83) compared to the high-stable group than individuals who were current or former smokers. Non-drinkers had also increased odds of being in the decliners or in the low-stable group (OR:1.21; 95%CI:1.05–1.39, OR:1.62; 95%CI:1.14–2.30, respectively) compared to the high-stable group. Membership in the moderate-stable group was not significantly associated with any of the examined lifestyle behaviours.

**Table 3:** Estimates and standard errors for the four-class growth mixture model of health.

		Class 1-Decliners (n = 3,161)	Class 2-Moderate-stable (n = 1,824)	Class 3-High-stable (n = 8,247)	Class 4-Low-stable (n = 756)
		Estimate (SE)			
**Intercept**	Mean	68.27 (0.40)**	56.39 (1.31)**	75.83 (0.29)**	39.69 (1.32)**
**Slope**	Mean	−28.29 (1.31)**	−7.71 (1.37)**	−11.52 (0.31)**	−6.46 (6.94)
**Variance-Covariance**	Intercept	25.05 (2.38)**			
	Slope	20.44 (3.77)**			
	Intercept-Slope	17.05 (3.10)**			
**Logit Coefficients**^**†**^		**Estimate (SE)**			
Physical Activity (Non-physically active vs active)		0.33 (0.09)**	−0.06 (0.28)	Reference	2.17 (0.41)**
Ever smoking (Never smokers vs current/former smokers)		−0.39 (0.09)**	−0.28 (0.19)	Reference	−0.58 (0.20)**
Drinking of alcohol (Never and no drinkers vs drinkers)		0.19 (0.07)**	0.04 (0.17)	Reference	0.48 (0.18)**
**Odds Ratio (95%CI)**					
Physical Activity (Non-physically active vs active)		1.39 (1.17–1.66)	0.94 (0.54–1.63)	Reference	8.78 (3.92–19.56)
Ever smoking (Never smokers vs current/former smokers)		0.68 (0.57–0.81)	0.76 (0.52–1.10)	Reference	0.56 (0.38–0.83)
Drinking of alcohol (Never and no drinkers vs drinkers)		1.21 (1.05–1.39)	1.04 (0.75–1.45)	Reference	1.62 (1.14–2.30)

Notes. SE: standard errors. **statistically significant in 0.05 level; *statistically significant in 0.10 level; ^†^adjusted for sex, age and education level.

#### Lifestyle behaviour as predictors of growth factors

Supplementary Table S9 presents the coefficients for the regression of each class specific growth factors on the lifestyle factors adjusted for socio-demographics. Only physical activity from the lifestyle behaviour covariates was associated with the baseline health score in the decliners group. More specifically, non-physically active participants were associated with lower baseline scores. In the other groups (i.e. moderate-stable, high-stable, low-stable) the average baseline health score was not significantly associated with physical activity, smoking or alcohol consumption. Regarding the rate of change in the decliners group and the high-stable group, non-drinking was associated with higher rates of deterioration. On the contrary, in the low-stable group alcohol abstinence was associated with lower rates of deterioration. Lifestyle covariates were not associated with the slope in the moderate-stable group, whereas the average rate of change in the low-stable class was not significant.

## Discussion

Based on a representative sample of older adults from Mexico, we generated a metric of health status in older age incorporating multiple dimensions of functioning measures and identified different groups of ageing trajectories across 14 years of follow-up. The findings in our study are consistent with previous research indicating that health outcome in older life is quite a heterogeneous process^45–48^. In our dataset, we identified four distinct latent trajectories of health which we named as follows: “decliners”, “moderate-stable”, “high-stable” and “low-stable”. Decliners are people who started with a high level of health in the baseline but exhibited the worst decline in the follow-up waves and a high death probability. The moderate-stable class had those participants starting in a moderate level and continue within a moderate level; this group could represent the usual agers. On the contrary, the high-stable group includes individuals who started high and concluded with a high level of health after 14 years of follow-up and the lowest death probability. This group could be characterised as the one exhibiting the ideal ageing trajectory. Finally, the low-stable class, representing the unhealthy agers, encompasses those participants who started low and finished low and exhibited the greatest probability of death in 2015.

Our findings regarding the number of distinct trajectories agree in general with research in high-income countries, even though a unanimous consensus regarding the number of identified latent classes is lacking. For instance, a study with data (n = 798; 9 years of follow-up) from the InCHIANTI cohort (Italy) and the Longitudinal Aging Study Amsterdam (LASA) suggested three different trajectories in the functional decline status of people 60–70 years old (no/little decline, intermediate decline, severe decline)^46^. Three distinct trajectories of ageing well (stable-good ageing well; initially ageing well then deteriorating; stable-poor) were also identified in a sample (n = 1,000; 16 years of follow-up) from the Melbourne Longitudinal Studies on Healthy Ageing (MELSHA)^45^. On the contrary and in agreement with our findings, Hsu and Jones (2012) identified four distinct trajectories (successful ageing; usual aging; health declining; and care demanding) in a study with older Taiwanese^49^. Three to four distinct trajectories were also identified in another LASA study where the focus was the cognitive and functional indicator of successful ageing, respectively^50^. Variety in the operational definitions of health outcomes in older age, differences in the study designs (i.e. sample size, follow-up time) seem to considerably impact the number of the identified trajectories.

In our analyses, participants with history of physical inactivity, smoking and alcohol abstinence were associated with an increased risk of accelerated decline in health and consistent poor health during the ageing process. These findings are in accordance with other studies indicating the beneficial effect of healthy lifestyle behaviour for a better ageing^8,51,52^. Physical activity was the strongest marker of the classes with non-physically active participants being almost 9 times more likely to be in the low-stable group compare to the high-stable group. Other studies using Mexican cohorts have also indicated that physical activity is associated with a decreased risk of cognitive decline and disability^53,54^. Our study replicated the finding that smoking abstinence is beneficially associated with better health outcomes in later life^8,13^. Regarding alcohol consumption, we found that it is a marker of better ageing trajectories in accordance with other studies reporting a beneficial association of light alcohol consumption with reduced risk of functional health decline^55^. However, these findings should be interpreted with caution as there are contradictory findings regarding the beneficial effect of limited alcohol consumption^56,57^.

To the best of our knowledge, this is the first study that examined healthy ageing in a longitudinal framework in a representative older cohort of Mexican people. The benefit of the conditional GMM approach is that it can identify these latent classes which indicate a consistent low functioning or a steep deterioration. Hence, by this way it can help researchers and policymakers to focus on those who are in risk and target them for future interventions. From a methodological point of view, the implementation of a one-step approach in the estimation of the GMM, which allowed the simultaneous incorporation of covariates, provides a more precise estimation of the covariates effects as class memberships are treated as latent variables and thus findings are less prone to measurement error^58^.

Among the strengths of our study is the use of a Bayesian multilevel IRT model. To better capture the underlying variable of health in old age, we employed a measurement model and then used this as an outcome in our GMM analyses. This approach allowed us to have different sets of questions per wave by also taking into account between and within (across the waves) participants information and simultaneously estimate all parameters^26^. The biggest strength of this model is that the latent construct of health was estimated in a multilevel framework allowing item parameters to vary across waves, whereas a common measurement of health was preserved;^23^ this feature allowed for the comparison of health metric among waves. Our measurement approach also contributed to the operationalisation of health in older age on a continuum, avoiding the often employed but unrealistic threshold approach (i.e. dichotomising participants as healthy or non-healthy agers)^59^. Additionally, our study is among the first ones that employed functional ability items to operationalise healthy ageing in accordance with the WHO framework^17^. Similar methodologies have recently been adopted in studies employing data from cohorts in the UK and the USA^48,60^.

Limitations of this study include the high attrition rate occurred during the 14 years of follow-up. In our models we assumed MAR mechanism, however as in all longitudinal studies of older people, there is a significant attrition due to death creating a survival bias towards healthier people. In addition, our analyses focused on people 50 years old and over without considering early life exposures. Nevertheless, a review has indicated the considerable impact of early life factors and events to health outcomes in older age^61^. Furthermore, since we only adjusted for age, gender and education level, the impact of lifestyle behaviours on the trajectories may be contributed to other unadjusted confounding factors. Additionally, as all information was self-reported measurement errors could not be excluded. Another limitation of our study is the way lifestyle behaviour variables were measured. The questions about physical activity and alcohol were too broad not allowing to assess the impact of different frequencies and intensities on the ageing trajectories. In particular, we could not identify former-excessive drinkers and investigate the impact of alcohol abuse in early life on healthy ageing.

Our study focused on distal lifestyle predictors early in mid-life to identify opportunities for health maintenance as people growing older. However, we also know that reverse causality could also be an issue, especially for physical activity (i.e. better health as people age could also affect physical activity levels). As a result, future research should focus on time-varying measures of physical activity that could help us to investigate the direction of these causal pathways. Furthermore, a more precise and objective measurement of physical activity and alcohol consumption could contribute to specifically identify the quantities that mostly improve or deteriorate health in older populations. In addition, even though, GMM is a sensitive approach able to identify latent subpopulations, it is data-driven and hugely dependable on the variation and characteristics of the sample. Future research should also focus on replicating these findings and advance the current knowledge in the field, even though comparability among cohorts is challenging. Finally, including younger cohorts in the analyses will contribute to a life-course perspective investigation and to examine whether similar trajectories are also observed^62,63^.

In conclusion, our findings show that older Mexicans age by following different trajectories of health and that lifestyle behaviours play an important role in these developmental processes. Physical activity and smoking abstinence are associated with better ageing trajectories, as well as the non-alcohol avoidance. In accordance with previous research, our results highlight the need for health policies and prevention strategies in the area^11^. Establishing non-pharmacological interventions that promote the adoption of a healthy lifestyle from early on could benefit older people to increase the number of years spent in a good health. In addition, it will assist governments and societies to more effectively deal with the public health burden. This is particularly important as Mexico will face a dramatic ageing population growth in the following years.

### Ethical approval

The MHAS study protocol and instruments were approved by the Institutional Review Board or Ethics Committee of the University of Texas Medical Branch, the Instituto Nacional de Estadística y Geografía (INEGI) in Mexico, and the Instituto Nacional de Salud Pública (INSP) in Mexico^19,20^. All selected subjects signed informed consent when the study started and were free to refuse participation in the study. All surveys completed by INEGI follow standard procedures to ensure respondent confidentiality and privacy of information in accordance to the ethical standards of INEGI and with the Helsinki ethical standards^64^.

## Supplementary information

supplementary material

## Data Availability

The data analysed during the current study were obtained by the official website of the Mexican Health and Aging Study. Codes for the statistical analyses are available from the corresponding author on reasonable request.
