# Supplementary material for: Healthy ageing trajectories and lifestyle behaviour: the Mexican Health and Aging Study
Source: Sci Rep. 2019 Jul 30;9:11041. doi: 10.1038/s41598-019-47238-w (PMC6667468; doi:10.1038/s41598-019-47238-w)
Supplement: Supplementary file 1 — supplementary material [file 41598_2019_47238_MOESM1_ESM.docx]

**Healthy ageing trajectories and lifestyle behaviour: the Mexican Health and Aging Study**

Christina Daskalopoulou; Artemis Koukounari; Yu-Tzu Wu; Graciela Muniz Terrera; Francisco Félix Caballero; Javier de la Fuente; Stefanos Tyrovolas; Demosthenes B. Panagiotakos; Martin Prince and Matthew Prina.

**Supplementary Table S1: Items/ Questions per wave**

| Items/ Wave | 2001 | 2003 | 2012 | 2015 |
| --- | --- | --- | --- | --- |
| Because of a health problem, do you have difficulty with walking several blocks? | x | x | x | x |
| Because of a health problem, do you have difficulty with walking one block? | x | x | x | x |
| Because of a health problem, do you have difficulty with sitting for about 2 hours? | x | x | x | x |
| Because of a health problem, do you have difficulty with getting up from a chair after sitting for long periods? | x | x | x | x |
| Because of a health problem, do you have difficulty with climbing several flights of stairs without resting? | x | x | x | x |
| Because of a health problem, do you have difficulty climbing one flight of stairs without resting? | x | x | x | x |
| Because of a health problem, do you have difficulty with stooping, kneeling, or crouching? | x | x | x | x |
| Because of a health problem, do you have difficulty with reaching or extending your arms above shoulder level? | x | x | x | x |
| Because of a health problem, do you have difficulty with pulling or pushing large objects like a living-room chair? | x | x | x | x |
| Because of a health problem, do you have difficulty with lifting or carrying objects weighting over 5 kg, like a heavy bag of groceries? | x | x | x | x |
| Because of a health problem, do you have difficulty with picking up a 1 peso coin from the table? | x | x | x | x |
| Because of a health problem, do you have difficulty with dressing including putting on shoes and socks? | x | x | x | x |
| Because of a health problem, do you have any difficulty with walking across a room? | x | x | x | x |
| Because of a health problem, do you have any difficulty with bathing or showering? | x | x | x | x |
| Because of a health problem, do you have any difficulty with eating, such as cutting your food? | x | x | x | x |
| Because of a health problem, do you have any difficulty with getting in or out of bed? | x | x | x | x |
| Because of a health problem, do you have any difficulty with using the toilet, including getting on and off the toilet or squatting? | x | x | x | x |
| Because of a health problem, do you have any difficulty with preparing a hot meal? | x | x | x | x |
| Because of a health problem, do you have any difficulty with shopping for groceries? | x | x | x | x |
| Because of a health problem, do you have any difficulty with taking medications (if you take any or needed to do so)? | x | x | x | x |
| Because of a health problem, do you have any difficulty managing your money? | x | x | x | x |
| How is your vision (using glasses)? Is it... | x | x | x | x |
| How is you hearing/auditory range (using hearing aid or auditory device)? | x | x |  | x |
| Do you often suffer from pain? | x | x | x | x |
| These questions refer to how you have felt during the past week. For each question please tell me if the majority of the time: You felt that everything you did was an effort | x | x | x | x |
| You felt your sleep was restless | x | x | x | x |
| You felt you had a lot of energy | x | x | x | x |
| During the last two years have you frequently had any of involuntary loss of urine? | x | x | x | x |
| Copying of figures 2001-2003 | x | x |  |  |
| Copying of figures 2012-2015 |  |  | x | x |
| Visual scanning | x | x | x | x |
| Visual memory 2001-2003 | x | x |  |  |
| Visual memory 2012-2015 |  |  | x | x |
| Days-orientation |  | x | x | x |
| Months-orientation |  | x | x | x |
| Years-orientation |  | x | x | x |
| Verbal learning | x | x | x | x |
| Verbal recall | x | x | x | x |
| Verbal fluency |  |  | x | x |
| Numeracy |  |  | x | x |

Notes: Copying of figures 2001-2003: respondent’s ability to copy two intact figures; Copying of figures 2012-2015: respondent’s ability to copy one figure; Visual scanning: measured by asking participants to circle all figures that are identical to a specific stimulus shown previously within an array of different stimuli (a total of 60 objects; the respondent passes the visual scanning with a grade greater than 24); Visual memory 2001-2003: measured by asking respondents to remember the figures they copied and to draw them on a blank piece of paper; Visual memory 2012-2015: measured by asking respondents to remember the figure they copied and to draw it on a blank piece of paper; Verbal learning: measured by asking participants to listen to a list of 8 words and repeat them; Verbal recall: measured by asking participants to repeat as many of the words they remember form the list provided in the verbal learning task; Verbal fluency: number of animals excluding the ones repeated that the participant could name in one minute; Numeracy: participants were asked to count backward form 20 to 0, when they asked for a 2^nd^ attempt the results of the 2^nd^ attempt was taken into account.

**Results of the Bayesian multilevel item response theory model**

**Model 1**: no variance in difficulty and discrimination parameters (σ_b,i_^2^ and σ_a,i_^2^ were assumed to be zero). Number of iterations: 7,000; Number of burn-in: 100. DIC: 10,642,623; EAP reliability: 0.851.

**Supplementary Table S2: Results of Model 1**

| Model 1: | | | | | |
| --- | --- | --- | --- | --- | --- |
| parameter | Mean | Standard deviation | R-hat | Q5 | Q95 |
| b[1] | -0.713 | 0.007 | 1.000 | -0.725 | -0.701 |
| b[2] | -1.398 | 0.009 | 1.000 | -1.413 | -1.384 |
| b[3] | -1.080 | 0.008 | 1.010 | -1.093 | -1.066 |
| b[4] | -0.653 | 0.007 | 1.000 | -0.665 | -0.641 |
| b[5] | -0.258 | 0.007 | 1.000 | -0.269 | -0.246 |
| b[6] | -0.911 | 0.008 | 1.000 | -0.924 | -0.898 |
| b[7] | -0.292 | 0.007 | 1.000 | -0.304 | -0.282 |
| b[8] | -1.413 | 0.009 | 1.000 | -1.427 | -1.397 |
| b[9] | -0.900 | 0.008 | 1.000 | -0.912 | -0.887 |
| b[10] | -0.922 | 0.008 | 1.000 | -0.934 | -0.909 |
| b[11] | -1.865 | 0.011 | 1.000 | -1.883 | -1.846 |
| b[12] | -1.721 | 0.010 | 1.000 | -1.738 | -1.705 |
| b[13] | -2.013 | 0.012 | 1.000 | -2.032 | -1.993 |
| b[14] | -2.270 | 0.015 | 1.010 | -2.294 | -2.246 |
| b[15] | -2.611 | 0.017 | 1.010 | -2.640 | -2.582 |
| b[16] | -1.941 | 0.012 | 1.000 | -1.961 | -1.922 |
| b[17] | -2.184 | 0.014 | 1.000 | -2.206 | -2.161 |
| b[18] | -2.200 | 0.014 | 1.000 | -2.223 | -2.178 |
| b[19] | -1.807 | 0.011 | 1.000 | -1.825 | -1.789 |
| b[20] | -2.423 | 0.016 | 1.010 | -2.449 | -2.398 |
| b[21] | -2.482 | 0.016 | 1.020 | -2.508 | -2.455 |
| b[22] | -1.609 | 0.010 | 1.000 | -1.625 | -1.592 |
| b[23] | -1.960 | 0.013 | 1.000 | -1.981 | -1.938 |
| b[24] | -0.289 | 0.007 | 1.020 | -0.300 | -0.277 |
| b[25] | -0.395 | 0.007 | 1.000 | -0.406 | -0.384 |
| b[26] | -0.299 | 0.007 | 1.000 | -0.310 | -0.288 |
| b[27] | 0.204 | 0.007 | 1.000 | 0.192 | 0.214 |
| b[28] | -1.383 | 0.009 | 1.000 | -1.398 | -1.369 |
| b[29] | -0.616 | 0.010 | 1.000 | -0.633 | -0.600 |
| b[30] | -0.821 | 0.012 | 1.000 | -0.841 | -0.801 |
| b[31] | 0.324 | 0.007 | 1.000 | 0.312 | 0.335 |
| b[32] | 0.585 | 0.009 | 1.000 | 0.571 | 0.600 |
| b[33] | -0.035 | 0.012 | 1.000 | -0.055 | -0.016 |
| b[34] | -0.736 | 0.009 | 1.010 | -0.751 | -0.721 |
| b[35] | -1.596 | 0.011 | 1.010 | -1.615 | -1.578 |
| b[36] | -1.031 | 0.010 | 1.000 | -1.047 | -1.015 |
| b[37] | -0.130 | 0.007 | 1.000 | -0.140 | -0.119 |
| b[38] | -0.676 | 0.007 | 1.000 | -0.688 | -0.664 |
| b[39] | -1.072 | 0.012 | 1.000 | -1.092 | -1.053 |
| b[40] | -1.543 | 0.015 | 1.000 | -1.567 | -1.519 |
| sigma1 | 0.720 | 0.003 | 1.010 | 0.715 | 0.725 |
| sigma2 | 0.263 | 0.111 | 1.000 | 0.148 | 0.450 |
| mu.b | -1.131 | 0.139 | 1.000 | -1.359 | -0.901 |
| omega.b | 0.859 | 0.099 | 1.000 | 0.715 | 1.034 |

**Model 2**: item-specific difficulty variance and no variance in the discrimination parameters (σ_b,i_^2^ is estimated). Number of iterations: 7,000; Number of burn-in: 100. DIC: 10,631,457; EAP reliability: 0.849.

**Supplementary Table S3: Results of Model 2**

| Model 2 | | | | | |
| --- | --- | --- | --- | --- | --- |
| parameter | Mean | Standard Deviation | R-hat | Q5 | Q95 |
| b[1] | -0.702 | 0.010 | 1.270 | -0.718 | -0.685 |
| b[2] | -1.393 | 0.011 | 1.200 | -1.411 | -1.374 |
| b[3] | -1.081 | 0.010 | 1.300 | -1.097 | -1.065 |
| b[4] | -0.644 | 0.009 | 1.320 | -0.658 | -0.628 |
| b[5] | -0.249 | 0.009 | 1.380 | -0.263 | -0.234 |
| b[6] | -0.901 | 0.010 | 1.290 | -0.917 | -0.884 |
| b[7] | -0.270 | 0.009 | 1.350 | -0.285 | -0.254 |
| b[8] | -1.410 | 0.011 | 1.190 | -1.428 | -1.391 |
| b[9] | -0.888 | 0.010 | 1.310 | -0.903 | -0.871 |
| b[10] | -0.908 | 0.009 | 1.260 | -0.923 | -0.893 |
| b[11] | -1.866 | 0.012 | 1.150 | -1.886 | -1.846 |
| b[12] | -1.722 | 0.012 | 1.210 | -1.741 | -1.702 |
| b[13] | -2.014 | 0.013 | 1.110 | -2.035 | -1.994 |
| b[14] | -2.277 | 0.016 | 1.110 | -2.302 | -2.252 |
| b[15] | -2.624 | 0.018 | 1.060 | -2.654 | -2.592 |
| b[16] | -1.947 | 0.013 | 1.110 | -1.968 | -1.925 |
| b[17] | -2.191 | 0.016 | 1.130 | -2.217 | -2.167 |
| b[18] | -2.202 | 0.016 | 1.160 | -2.228 | -2.177 |
| b[19] | -1.807 | 0.012 | 1.160 | -1.827 | -1.787 |
| b[20] | -2.433 | 0.017 | 1.180 | -2.461 | -2.406 |
| b[21] | -2.487 | 0.018 | 1.090 | -2.516 | -2.458 |
| b[22] | -1.640 | 0.012 | 1.230 | -1.660 | -1.621 |
| b[23] | -2.011 | 0.122 | 2.530 | -2.235 | -1.840 |
| b[24] | -0.322 | 0.009 | 1.310 | -0.337 | -0.306 |
| b[25] | -0.418 | 0.009 | 1.270 | -0.433 | -0.402 |
| b[26] | -0.304 | 0.009 | 1.310 | -0.319 | -0.288 |
| b[27] | 0.167 | 0.009 | 1.290 | 0.153 | 0.182 |
| b[28] | -1.379 | 0.010 | 1.240 | -1.397 | -1.362 |
| b[29] | -0.655 | 0.086 | 1.210 | -0.771 | -0.509 |
| b[30] | -0.675 | 0.096 | 1.890 | -0.839 | -0.528 |
| b[31] | 0.312 | 0.010 | 1.400 | 0.297 | 0.328 |
| b[32] | 0.389 | 0.115 | 2.500 | 0.173 | 0.550 |
| b[33] | -0.026 | 0.127 | 2.350 | -0.251 | 0.185 |
| b[34] | -0.790 | 0.100 | 1.850 | -0.971 | -0.664 |
| b[35] | -1.524 | 0.077 | 2.770 | -1.652 | -1.394 |
| b[36] | -0.780 | 0.106 | 1.840 | -0.964 | -0.623 |
| b[37] | -0.142 | 0.009 | 1.380 | -0.157 | -0.127 |
| b[38] | -0.659 | 0.009 | 1.270 | -0.674 | -0.643 |
| b[39] | -1.008 | 0.115 | 1.180 | -1.156 | -0.731 |
| b[40] | -1.335 | 0.074 | 1.060 | -1.448 | -1.204 |
| sigma1 | 0.731 | 0.003 | 1.020 | 0.726 | 0.737 |
| sigma2 | 0.270 | 0.111 | 1.000 | 0.153 | 0.472 |
| mu.b | -1.125 | 0.133 | 1.010 | -1.336 | -0.906 |
| omega.b | 0.847 | 0.096 | 1.000 | 0.706 | 1.012 |
| sigma.b[1] | 0.524 | 0.214 | 1.000 | 0.308 | 0.897 |
| sigma.b[2] | 0.548 | 0.256 | 1.000 | 0.302 | 0.944 |
| sigma.b[3] | 0.535 | 0.213 | 1.000 | 0.300 | 0.932 |
| sigma.b[4] | 0.551 | 0.308 | 1.000 | 0.307 | 0.937 |
| sigma.b[5] | 0.526 | 0.205 | 1.000 | 0.303 | 0.903 |
| sigma.b[6] | 0.552 | 0.265 | 1.000 | 0.310 | 0.977 |
| sigma.b[7] | 0.544 | 0.233 | 1.000 | 0.309 | 0.938 |
| sigma.b[8] | 0.547 | 0.240 | 1.000 | 0.304 | 0.940 |
| sigma.b[9] | 0.544 | 0.227 | 1.000 | 0.305 | 0.945 |
| sigma.b[10] | 0.552 | 0.266 | 1.000 | 0.314 | 0.961 |
| sigma.b[11] | 0.542 | 0.211 | 1.000 | 0.308 | 0.963 |
| sigma.b[12] | 0.557 | 0.229 | 1.000 | 0.314 | 1.024 |
| sigma.b[13] | 0.539 | 0.211 | 1.000 | 0.306 | 0.936 |
| sigma.b[14] | 0.545 | 0.220 | 1.000 | 0.310 | 0.941 |
| sigma.b[15] | 0.562 | 0.246 | 1.000 | 0.308 | 1.008 |
| sigma.b[16] | 0.549 | 0.218 | 1.000 | 0.311 | 0.990 |
| sigma.b[17] | 0.553 | 0.238 | 1.000 | 0.309 | 0.951 |
| sigma.b[18] | 0.534 | 0.206 | 1.000 | 0.303 | 0.890 |
| sigma.b[19] | 0.575 | 0.257 | 1.000 | 0.315 | 1.025 |
| sigma.b[20] | 0.545 | 0.238 | 1.000 | 0.304 | 0.964 |
| sigma.b[21] | 0.542 | 0.234 | 1.000 | 0.313 | 0.956 |
| sigma.b[22] | 0.562 | 0.242 | 1.000 | 0.320 | 1.033 |
| sigma.b[23] | 0.582 | 0.250 | 1.010 | 0.325 | 0.998 |
| sigma.b[24] | 0.560 | 0.216 | 1.010 | 0.324 | 0.963 |
| sigma.b[25] | 0.546 | 0.224 | 1.000 | 0.310 | 0.959 |
| sigma.b[26] | 0.543 | 0.239 | 1.000 | 0.298 | 1.018 |
| sigma.b[27] | 0.576 | 0.242 | 1.000 | 0.326 | 0.995 |
| sigma.b[28] | 0.585 | 0.241 | 1.000 | 0.329 | 1.033 |
| sigma.b[29] | 0.562 | 0.222 | 1.010 | 0.321 | 0.983 |
| sigma.b[30] | 0.641 | 0.263 | 1.140 | 0.351 | 1.159 |
| sigma.b[31] | 0.677 | 0.285 | 1.000 | 0.379 | 1.177 |
| sigma.b[32] | 0.668 | 0.280 | 1.010 | 0.365 | 1.194 |
| sigma.b[33] | 0.610 | 0.258 | 1.000 | 0.337 | 1.082 |
| sigma.b[34] | 0.592 | 0.256 | 1.020 | 0.327 | 1.061 |
| sigma.b[35] | 0.576 | 0.239 | 1.010 | 0.321 | 1.036 |
| sigma.b[36] | 0.681 | 0.292 | 1.050 | 0.361 | 1.242 |
| sigma.b[37] | 0.549 | 0.257 | 1.000 | 0.308 | 0.993 |
| sigma.b[38] | 0.584 | 0.238 | 1.000 | 0.332 | 1.005 |
| sigma.b[39] | 0.681 | 0.291 | 1.170 | 0.367 | 1.207 |
| sigma.b[40] | 0.640 | 0.278 | 1.090 | 0.346 | 1.139 |

**Model 3**: homogeneous difficulty variance and no discrimination variance (a joint variance of all item difficulties is estimated σ_b,1_^2^= σ_b,2_^2^=…= σ_b,I_^2^). Number of iterations: 7,000; Number of burn-in: 100. DIC: 10,631,464; EAP reliability: 0.854.

**Supplementary Table S4: Results of Model 3**

| Model 3 | | | | | |
| --- | --- | --- | --- | --- | --- |
| parameter | Mean | Standard Deviation | R-hat | Q5 | Q95 |
| b[1] | -0.711 | 0.008 | 1.090 | -0.724 | -0.697 |
| b[2] | -1.403 | 0.009 | 1.040 | -1.417 | -1.387 |
| b[3] | -1.091 | 0.008 | 1.030 | -1.105 | -1.077 |
| b[4] | -0.654 | 0.008 | 1.050 | -0.667 | -0.640 |
| b[5] | -0.260 | 0.008 | 1.100 | -0.273 | -0.248 |
| b[6] | -0.910 | 0.009 | 1.040 | -0.924 | -0.895 |
| b[7] | -0.280 | 0.008 | 1.060 | -0.293 | -0.268 |
| b[8] | -1.419 | 0.009 | 1.040 | -1.434 | -1.405 |
| b[9] | -0.898 | 0.009 | 1.090 | -0.912 | -0.884 |
| b[10] | -0.918 | 0.008 | 1.060 | -0.931 | -0.904 |
| b[11] | -1.874 | 0.011 | 1.040 | -1.892 | -1.855 |
| b[12] | -1.731 | 0.011 | 1.040 | -1.749 | -1.713 |
| b[13] | -2.023 | 0.012 | 1.030 | -2.043 | -2.003 |
| b[14] | -2.287 | 0.015 | 1.020 | -2.310 | -2.263 |
| b[15] | -2.634 | 0.018 | 1.040 | -2.663 | -2.604 |
| b[16] | -1.956 | 0.012 | 1.020 | -1.976 | -1.938 |
| b[17] | -2.203 | 0.014 | 1.050 | -2.226 | -2.179 |
| b[18] | -2.211 | 0.014 | 1.030 | -2.234 | -2.188 |
| b[19] | -1.816 | 0.012 | 1.050 | -1.835 | -1.797 |
| b[20] | -2.441 | 0.015 | 1.010 | -2.465 | -2.416 |
| b[21] | -2.498 | 0.017 | 1.050 | -2.526 | -2.472 |
| b[22] | -1.649 | 0.010 | 1.070 | -1.665 | -1.633 |
| b[23] | -1.991 | 0.047 | 1.150 | -2.072 | -1.923 |
| b[24] | -0.332 | 0.008 | 1.110 | -0.344 | -0.319 |
| b[25] | -0.428 | 0.007 | 1.060 | -0.440 | -0.416 |
| b[26] | -0.315 | 0.007 | 1.060 | -0.327 | -0.303 |
| b[27] | 0.157 | 0.008 | 1.090 | 0.144 | 0.169 |
| b[28] | -1.389 | 0.009 | 1.020 | -1.404 | -1.373 |
| b[29] | -0.624 | 0.082 | 1.410 | -0.760 | -0.482 |
| b[30] | -0.852 | 0.079 | 1.050 | -0.970 | -0.707 |
| b[31] | 0.303 | 0.008 | 1.060 | 0.290 | 0.316 |
| b[32] | 0.567 | 0.072 | 1.470 | 0.422 | 0.658 |
| b[33] | -0.084 | 0.068 | 1.440 | -0.208 | 0.010 |
| b[34] | -0.768 | 0.044 | 1.430 | -0.842 | -0.690 |
| b[35] | -1.588 | 0.046 | 1.090 | -1.666 | -1.513 |
| b[36] | -0.986 | 0.051 | 1.310 | -1.055 | -0.876 |
| b[37] | -0.152 | 0.008 | 1.090 | -0.164 | -0.139 |
| b[38] | -0.669 | 0.008 | 1.080 | -0.683 | -0.655 |
| b[39] | -1.082 | 0.078 | 1.040 | -1.210 | -0.942 |
| b[40] | -1.570 | 0.081 | 1.780 | -1.700 | -1.412 |
| sigma1 | 0.732 | 0.003 | 1.000 | 0.726 | 0.737 |
| sigma2 | 0.283 | 0.112 | 1.000 | 0.161 | 0.513 |
| mu.b | -1.137 | 0.131 | 1.010 | -1.354 | -0.932 |
| omega.b | 0.858 | 0.102 | 1.000 | 0.710 | 1.041 |
| sigma.b | 0.160 | 0.011 | 1.080 | 0.145 | 0.179 |

Model 4: variance in difficulty and discrimination parameters and estimation of the hyperprior distribution (σ_b,i_^2^ and σ_a,i_^2^ are estimated and hyperpriors with parameters μ_σ_, ω_b_, ω_a_). Number of iterations: 7,000; Number of burn-in: 100. DIC: 10,572,560; EAP reliability: 0.839.

**Supplementary Table S5: Results of Model 4**

| Model 4 | | | | | | |  |
| --- | --- | --- | --- | --- | --- | --- | --- |
| parameter | | Mean | Standard Deviation | R-hat | Q5 | Q95 |  |
| b[1] | -1.178 | | 0.023 | 1.640 | -1.216 | -1.140 | |
| b[2] | -2.271 | | 0.037 | 1.060 | -2.330 | -2.208 | |
| b[3] | -1.256 | | 0.013 | 1.150 | -1.279 | -1.236 | |
| b[4] | -0.883 | | 0.013 | 1.240 | -0.904 | -0.863 | |
| b[5] | -0.342 | | 0.013 | 1.820 | -0.365 | -0.322 | |
| b[6] | -1.409 | | 0.020 | 1.450 | -1.444 | -1.378 | |
| b[7] | -0.427 | | 0.012 | 1.560 | -0.448 | -0.408 | |
| b[8] | -1.656 | | 0.016 | 1.000 | -1.683 | -1.629 | |
| b[9] | -1.319 | | 0.017 | 1.210 | -1.349 | -1.292 | |
| b[10] | -1.321 | | 0.018 | 1.110 | -1.349 | -1.292 | |
| b[11] | -2.155 | | 0.023 | 1.000 | -2.192 | -2.117 | |
| b[12] | -2.413 | | 0.036 | 1.020 | -2.470 | -2.358 | |
| b[13] | -3.612 | | 0.118 | 1.500 | -3.738 | -3.368 | |
| b[14] | -3.910 | | 0.158 | 2.080 | -4.084 | -3.564 | |
| b[15] | -3.467 | | 0.101 | 1.470 | -3.594 | -3.314 | |
| b[16] | -2.938 | | 0.061 | 1.360 | -3.021 | -2.854 | |
| b[17] | -3.072 | | 0.074 | 1.650 | -3.166 | -2.936 | |
| b[18] | -2.968 | | 0.051 | 1.230 | -3.041 | -2.896 | |
| b[19] | -2.665 | | 0.042 | 1.050 | -2.733 | -2.609 | |
| b[20] | -2.863 | | 0.045 | 1.030 | -2.932 | -2.791 | |
| b[21] | -2.992 | | 0.055 | 1.140 | -3.057 | -2.886 | |
| b[22] | -1.452 | | 0.011 | 1.050 | -1.470 | -1.434 | |
| b[23] | -1.719 | | 0.070 | 1.580 | -1.828 | -1.600 | |
| b[24] | -0.309 | | 0.009 | 1.640 | -0.325 | -0.295 | |
| b[25] | -0.408 | | 0.010 | 1.480 | -0.425 | -0.393 | |
| b[26] | -0.265 | | 0.008 | 1.320 | -0.277 | -0.252 | |
| b[27] | 0.157 | | 0.008 | 1.370 | 0.144 | 0.170 | |
| b[28] | -1.234 | | 0.010 | 1.080 | -1.249 | -1.217 | |
| b[29] | -0.768 | | 0.117 | 2.690 | -0.934 | -0.533 | |
| b[30] | -1.062 | | 0.196 | 1.850 | -1.337 | -0.716 | |
| b[31] | 0.267 | | 0.007 | 1.070 | 0.255 | 0.279 | |
| b[32] | 0.109 | | 0.082 | 1.460 | -0.043 | 0.228 | |
| b[33] | 0.414 | | 0.182 | 3.310 | 0.163 | 0.676 | |
| b[34] | -0.515 | | 0.072 | 2.740 | -0.630 | -0.399 | |
| b[35] | -1.387 | | 0.048 | 1.220 | -1.457 | -1.298 | |
| b[36] | -1.088 | | 0.155 | 5.120 | -1.332 | -0.863 | |
| b[37] | -0.108 | | 0.007 | 1.250 | -0.120 | -0.097 | |
| b[38] | -0.554 | | 0.008 | 1.220 | -0.567 | -0.541 | |
| b[39] | -0.907 | | 0.078 | 1.020 | -1.022 | -0.779 | |
| b[40] | -1.423 | | 0.127 | 1.930 | -1.615 | -1.191 | |
| a[1] | 1.860 | | 0.034 | 1.490 | 1.807 | 1.919 | |
| a[2] | 1.896 | | 0.040 | 1.770 | 1.833 | 1.965 | |
| a[3] | 1.086 | | 0.019 | 1.650 | 1.054 | 1.115 | |
| a[4] | 1.382 | | 0.024 | 1.490 | 1.344 | 1.422 | |
| a[5] | 1.257 | | 0.022 | 1.520 | 1.223 | 1.292 | |
| a[6] | 1.721 | | 0.029 | 1.540 | 1.675 | 1.769 | |
| a[7] | 1.516 | | 0.025 | 1.380 | 1.475 | 1.553 | |
| a[8] | 1.132 | | 0.021 | 1.950 | 1.096 | 1.166 | |
| a[9] | 1.599 | | 0.028 | 1.570 | 1.556 | 1.646 | |
| a[10] | 1.547 | | 0.027 | 1.590 | 1.503 | 1.595 | |
| a[11] | 1.121 | | 0.025 | 1.850 | 1.078 | 1.160 | |
| a[12] | 1.558 | | 0.036 | 2.070 | 1.488 | 1.611 | |
| a[13] | 2.219 | | 0.100 | 2.590 | 2.012 | 2.339 | |
| a[14] | 2.120 | | 0.122 | 3.180 | 1.857 | 2.245 | |
| a[15] | 1.453 | | 0.072 | 2.160 | 1.333 | 1.546 | |
| a[16] | 1.750 | | 0.056 | 2.910 | 1.654 | 1.823 | |
| a[17] | 1.579 | | 0.065 | 2.960 | 1.453 | 1.665 | |
| a[18] | 1.505 | | 0.047 | 2.380 | 1.423 | 1.583 | |
| a[19] | 1.688 | | 0.035 | 1.510 | 1.631 | 1.744 | |
| a[20] | 1.194 | | 0.032 | 1.260 | 1.142 | 1.247 | |
| a[21] | 1.248 | | 0.046 | 1.890 | 1.159 | 1.307 | |
| a[22] | 0.457 | | 0.013 | 1.240 | 0.436 | 0.479 | |
| a[23] | 0.392 | | 0.083 | 2.580 | 0.244 | 0.512 | |
| a[24] | 0.654 | | 0.013 | 1.440 | 0.633 | 0.674 | |
| a[25] | 0.703 | | 0.013 | 1.480 | 0.681 | 0.723 | |
| a[26] | 0.470 | | 0.010 | 1.270 | 0.455 | 0.486 | |
| a[27] | 0.301 | | 0.008 | 1.180 | 0.287 | 0.315 | |
| a[28] | 0.474 | | 0.012 | 1.360 | 0.454 | 0.495 | |
| a[29] | 0.521 | | 0.110 | 1.840 | 0.283 | 0.665 | |
| a[30] | 0.439 | | 0.093 | 3.660 | 0.272 | 0.573 | |
| a[31] | 0.300 | | 0.009 | 1.090 | 0.286 | 0.316 | |
| a[32] | 0.340 | | 0.168 | 6.080 | 0.098 | 0.633 | |
| a[33] | 0.407 | | 0.089 | 1.070 | 0.284 | 0.547 | |
| a[34] | 0.268 | | 0.092 | 4.750 | 0.141 | 0.414 | |
| a[35] | 0.301 | | 0.095 | 2.830 | 0.106 | 0.440 | |
| a[36] | 0.289 | | 0.085 | 4.390 | 0.166 | 0.441 | |
| a[37] | 0.304 | | 0.009 | 1.150 | 0.290 | 0.319 | |
| a[38] | 0.295 | | 0.009 | 1.200 | 0.280 | 0.309 | |
| a[39] | 0.323 | | 0.103 | 2.470 | 0.170 | 0.495 | |
| a[40] | 0.328 | | 0.122 | 2.860 | 0.111 | 0.515 | |
| sigma1 | 0.964 | | 0.014 | 2.030 | 0.945 | 0.987 | |
| sigma2 | 0.253 | | 0.104 | 1.020 | 0.142 | 0.423 | |
| mu.b | -1.438 | | 0.192 | 1.000 | -1.743 | -1.112 | |
| omega.b | 1.182 | | 0.137 | 1.040 | 0.976 | 1.424 | |
| sigma.b[1] | 0.591 | | 0.235 | 1.010 | 0.334 | 1.011 | |
| sigma.b[2] | 0.600 | | 0.231 | 1.010 | 0.337 | 1.020 | |
| sigma.b[3] | 0.554 | | 0.256 | 1.010 | 0.313 | 0.996 | |
| sigma.b[4] | 0.583 | | 0.235 | 1.010 | 0.330 | 1.010 | |
| sigma.b[5] | 0.555 | | 0.238 | 1.000 | 0.309 | 1.018 | |
| sigma.b[6] | 0.607 | | 0.287 | 1.010 | 0.335 | 1.114 | |
| sigma.b[7] | 0.599 | | 0.247 | 1.010 | 0.332 | 1.079 | |
| sigma.b[8] | 0.564 | | 0.217 | 1.010 | 0.330 | 0.968 | |
| sigma.b[9] | 0.632 | | 0.313 | 1.020 | 0.348 | 1.126 | |
| sigma.b[10] | 0.653 | | 0.271 | 1.020 | 0.358 | 1.181 | |
| sigma.b[11] | 0.618 | | 0.326 | 1.000 | 0.344 | 1.066 | |
| sigma.b[12] | 0.634 | | 0.269 | 1.010 | 0.345 | 1.099 | |
| sigma.b[13] | 0.766 | | 0.339 | 1.120 | 0.394 | 1.404 | |
| sigma.b[14] | 0.843 | | 0.371 | 1.190 | 0.423 | 1.521 | |
| sigma.b[15] | 0.846 | | 0.372 | 1.040 | 0.453 | 1.541 | |
| sigma.b[16] | 0.703 | | 0.299 | 1.070 | 0.390 | 1.273 | |
| sigma.b[17] | 0.773 | | 0.346 | 1.080 | 0.413 | 1.362 | |
| sigma.b[18] | 0.625 | | 0.283 | 1.030 | 0.353 | 1.088 | |
| sigma.b[19] | 0.660 | | 0.269 | 1.000 | 0.369 | 1.130 | |
| sigma.b[20] | 0.633 | | 0.250 | 1.000 | 0.352 | 1.061 | |
| sigma.b[21] | 0.640 | | 0.268 | 1.000 | 0.360 | 1.139 | |
| sigma.b[22] | 0.539 | | 0.227 | 1.000 | 0.296 | 0.972 | |
| sigma.b[23] | 0.544 | | 0.215 | 1.000 | 0.311 | 0.935 | |
| sigma.b[24] | 0.542 | | 0.213 | 1.000 | 0.307 | 0.939 | |
| sigma.b[25] | 0.535 | | 0.213 | 1.000 | 0.307 | 0.934 | |
| sigma.b[26] | 0.532 | | 0.204 | 1.000 | 0.301 | 0.889 | |
| sigma.b[27] | 0.539 | | 0.221 | 1.000 | 0.303 | 0.947 | |
| sigma.b[28] | 0.601 | | 0.290 | 1.000 | 0.336 | 1.011 | |
| sigma.b[29] | 0.740 | | 0.348 | 1.290 | 0.374 | 1.452 | |
| sigma.b[30] | 0.746 | | 0.330 | 1.070 | 0.391 | 1.360 | |
| sigma.b[31] | 0.611 | | 0.235 | 1.000 | 0.351 | 1.071 | |
| sigma.b[32] | 0.799 | | 0.360 | 1.000 | 0.426 | 1.525 | |
| sigma.b[33] | 0.739 | | 0.350 | 1.130 | 0.378 | 1.352 | |
| sigma.b[34] | 0.554 | | 0.237 | 1.000 | 0.320 | 0.976 | |
| sigma.b[35] | 0.554 | | 0.235 | 1.000 | 0.306 | 0.970 | |
| sigma.b[36] | 0.745 | | 0.395 | 1.260 | 0.345 | 1.412 | |
| sigma.b[37] | 0.534 | | 0.219 | 1.000 | 0.301 | 0.926 | |
| sigma.b[38] | 0.601 | | 0.249 | 1.000 | 0.335 | 1.030 | |
| sigma.b[39] | 0.610 | | 0.250 | 1.040 | 0.331 | 1.096 | |
| sigma.b[40] | 0.704 | | 0.347 | 1.090 | 0.356 | 1.297 | |
| sigma.a[1] | 0.563 | | 0.226 | 1.000 | 0.314 | 0.977 | |
| sigma.a[2] | 0.532 | | 0.212 | 1.000 | 0.310 | 0.904 | |
| sigma.a[3] | 0.532 | | 0.210 | 1.000 | 0.311 | 0.937 | |
| sigma.a[4] | 0.555 | | 0.260 | 1.000 | 0.307 | 0.951 | |
| sigma.a[5] | 0.545 | | 0.223 | 1.000 | 0.311 | 0.996 | |
| sigma.a[6] | 0.553 | | 0.259 | 1.000 | 0.311 | 0.983 | |
| sigma.a[7] | 0.583 | | 0.267 | 1.000 | 0.323 | 1.060 | |
| sigma.a[8] | 0.539 | | 0.208 | 1.000 | 0.301 | 0.908 | |
| sigma.a[9] | 0.582 | | 0.266 | 1.000 | 0.324 | 1.015 | |
| sigma.a[10] | 0.588 | | 0.235 | 1.000 | 0.332 | 1.031 | |
| sigma.a[11] | 0.569 | | 0.228 | 1.000 | 0.322 | 0.991 | |
| sigma.a[12] | 0.539 | | 0.204 | 1.000 | 0.302 | 0.932 | |
| sigma.a[13] | 0.608 | | 0.269 | 1.000 | 0.342 | 1.061 | |
| sigma.a[14] | 0.671 | | 0.296 | 1.000 | 0.362 | 1.189 | |
| sigma.a[15] | 0.645 | | 0.256 | 1.020 | 0.357 | 1.132 | |
| sigma.a[16] | 0.563 | | 0.206 | 1.000 | 0.324 | 0.939 | |
| sigma.a[17] | 0.601 | | 0.243 | 1.000 | 0.346 | 1.012 | |
| sigma.a[18] | 0.561 | | 0.243 | 1.000 | 0.317 | 1.002 | |
| sigma.a[19] | 0.538 | | 0.220 | 1.000 | 0.313 | 0.938 | |
| sigma.a[20] | 0.553 | | 0.231 | 1.010 | 0.318 | 0.934 | |
| sigma.a[21] | 0.573 | | 0.237 | 1.000 | 0.322 | 1.049 | |
| sigma.a[22] | 0.529 | | 0.208 | 1.000 | 0.297 | 0.906 | |
| sigma.a[23] | 0.563 | | 0.249 | 1.000 | 0.310 | 0.983 | |
| sigma.a[24] | 0.529 | | 0.212 | 1.000 | 0.288 | 0.917 | |
| sigma.a[25] | 0.527 | | 0.214 | 1.000 | 0.300 | 0.955 | |
| sigma.a[26] | 0.525 | | 0.235 | 1.000 | 0.297 | 0.911 | |
| sigma.a[27] | 0.530 | | 0.211 | 1.000 | 0.304 | 0.940 | |
| sigma.a[28] | 0.536 | | 0.250 | 1.000 | 0.298 | 0.973 | |
| sigma.a[29] | 0.616 | | 0.253 | 1.010 | 0.351 | 1.088 | |
| sigma.a[30] | 0.581 | | 0.234 | 1.010 | 0.325 | 1.052 | |
| sigma.a[31] | 0.528 | | 0.202 | 1.000 | 0.307 | 0.938 | |
| sigma.a[32] | 0.626 | | 0.287 | 1.060 | 0.335 | 1.111 | |
| sigma.a[33] | 0.678 | | 0.305 | 1.070 | 0.362 | 1.283 | |
| sigma.a[34] | 0.572 | | 0.239 | 1.010 | 0.316 | 0.995 | |
| sigma.a[35] | 0.559 | | 0.254 | 1.010 | 0.312 | 1.023 | |
| sigma.a[36] | 0.586 | | 0.291 | 1.020 | 0.320 | 1.106 | |
| sigma.a[37] | 0.540 | | 0.236 | 1.000 | 0.297 | 0.976 | |
| sigma.a[38] | 0.523 | | 0.200 | 1.000 | 0.300 | 0.917 | |
| sigma.a[39] | 0.584 | | 0.264 | 1.010 | 0.325 | 1.029 | |
| sigma.a[40] | 0.571 | | 0.220 | 1.000 | 0.325 | 0.977 | |
| omega.a | 0.630 | | 0.081 | 1.340 | 0.505 | 0.770 | |

Model 3 showed the highest EAP reliability (0.854) and Model 4 the lowest DIC (10,572,560). Between those two we compared the EAP precision of measurement. Model 3 for most of the latent score range (between -3 and 3) exhibited errors below 0.5, whereas Model 4 indicated errors above 0.5 for latent scores above 0 (see **Supplementary** **Fig. 1** and **Fig. 2** below). In addition, the R-hat diagnostic of the MCMC algorithm showed values closer to 1 for Model 3. For these reasons we proceeded in our analyses with Model 3.

**Supplementary Figure 1: Precision of measurement-Model 3**

*Notes*: Spectrum of EAP (expected-a-posteriori) latent construct scores (initial range -3, 3) against standard deviation (SD)

**Supplementary Figure 2: Precision of measurement-Model 4**

*Notes*: Spectrum of EAP (expected-a-posteriori) latent construct scores (initial range -3, 3) against standard deviation (SD)

**Supplementary Table S6:** Correspondence of items b[1]-b[40] to the exact questions asked to participants.

| Parameter | Question |
| --- | --- |
| b[1] | Because of a health problem, do you have difficulty with walking several blocks? |
| b[2] | Because of a health problem, do you have difficulty with walking one block? |
| b[3] | Because of a health problem, do you have difficulty with sitting for about 2 hours? |
| b[4] | Because of a health problem, do you have difficulty with getting up from a chair after sitting for long periods? |
| b[5] | Because of a health problem, do you have difficulty with climbing several flights of stairs without resting? |
| b[6] | Because of a health problem, do you have difficulty climbing one flight of stairs without resting? |
| b[7] | Because of a health problem, do you have difficulty with stooping, kneeling, or crouching? |
| b[8] | Because of a health problem, do you have difficulty with reaching or extending your arms above shoulder level? |
| b[9] | Because of a health problem, do you have difficulty with pulling or pushing large objects like a living-room chair? |
| b[10] | Because of a health problem, do you have difficulty with lifting or carrying objects weighting over 5 kg, like a heavy bag of groceries? |
| b[11] | Because of a health problem, do you have difficulty with picking up a 1 peso coin from the table? |
| b[12] | Because of a health problem, do you have difficulty with dressing including putting on shoes and socks? |
| b[13] | Because of a health problem, do you have any difficulty with walking across a room? |
| b[14] | Because of a health problem, do you have any difficulty with bathing or showering? |
| b[15] | Because of a health problem, do you have any difficulty with eating, such as cutting your food? |
| b[16] | Because of a health problem, do you have any difficulty with getting in or out of bed? |
| b[17] | Because of a health problem, do you have any difficulty with using the toilet, including getting on and off the toilet or squatting? |
| b[18] | Because of a health problem, do you have any difficulty with preparing a hot meal? |
| b[19] | Because of a health problem, do you have any difficulty with shopping for groceries? |
| b[20] | Because of a health problem, do you have any difficulty with taking medications (if you take any or needed to do so)? |
| b[21] | Because of a health problem, do you have any difficulty managing your money? |
| b[22] | How is your vision (using glasses)? Is it... |
| b[23] | How is you hearing/auditory range (using hearing aid or auditory device)? |
| b[24] | Do you often suffer from pain? |
| b[25] | These questions refer to how you have felt during the past week. For each question please tell me if the majority of the time:  You felt that everything you did was an effort |
| b[26] | You felt your sleep was restless |
| b[27] | You felt you had a lot of energy |
| b[28] | During the last two years have you frequently had any of the following problems or inconveniences? Involuntary loss of urine |
| b[29] | Copying of figures 2001-2003 |
| b[30] | Copying of figures 2012-2015 |
| b[31] | Visual scanning |
| b[32] | Visual memory 2001-2003 |
| b[33] | Visual memory 2012-2015 |
| b[34] | Days-orientation |
| b[35] | Months-orientation |
| b[36] | Years-orientation |
| b[37] | Verbal learning |
| b[38] | Verbal recall |
| b[39] | Verbal fluency |
| b[40] | Numeracy |

**Supplementary Table S7: Exploratory Factor Analysis: Geomin factor loadings**

| Items/Questions | Factor1 | Factor2 | Factor3 | | Factor4 | |
| --- | --- | --- | --- | --- | --- | --- |
| Because of a health problem, do you have difficulty with walking several blocks? | 0.39 | **0.51** | 0.23 | 0.01 | |  |
| Because of a health problem, do you have difficulty with walking one block? | 0.49 | **0.63** | 0.04 | 0.03 | |  |
| Because of a health problem, do you have difficulty with climbing several flights of stairs without resting? | 0.03 | **0.55** | 0.49 | -0.04 | |  |
| Because of a health problem, do you have difficulty climbing one flight of stairs without resting? | 0.20 | **0.53** | 0.38 | 0.03 | |  |
| Because of a health problem, do you have difficulty with sitting for about 2 hours? | 0.04 | 0.05 | **0.78** | -0.04 | |  |
| Because of a health problem, do you have difficulty with getting up from a chair after sitting for long periods? | 0.09 | 0.09 | **0.80** | -0.08 | |  |
| Because of a health problem, do you have difficulty with stooping, kneeling, or crouching? | 0.30 | 0.18 | **0.53** | -0.08 | |  |
| Because of a health problem, do you have difficulty with reaching or extending your arms above shoulder level? | 0.40 | 0.04 | **0.43** | 0.01 | |  |
| Do you often suffer from pain? | -0.04 | 0.00 | **0.62** | 0.08 | |  |
| These questions refer to how you have felt during the past week. For each question please tell me if the majority of the time: You felt that everything you did was an effort | 0.03 | -0.15 | **0.72** | 0.19 | |  |
| You felt your sleep was restless | -0.12 | -0.21 | **0.73** | 0.13 | |  |
| You felt you had a lot of energy | -0.05 | 0.08 | **0.26** | 0.20 | |  |
| During the last two years have you frequently had any of the following problems or inconveniences? Involuntary loss of urine | 0.17 | 0.00 | **0.33** | 0.08 | |  |
| Because of a health problem, do you have difficulty with pulling or pushing large objects like a living-room chair? | **0.55** | 0.11 | 0.35 | -0.03 | |  |
| Because of a health problem, do you have difficulty with lifting or carrying objects weighting over 5 kg, like a heavy bag of groceries? | **0.56** | 0.09 | 0.33 | -0.04 | |  |
| Because of a health problem, do you have difficulty with picking up a 1 peso coin from the table? | **0.62** | 0.02 | 0.20 | 0.05 | |  |
| Because of a health problem, do you have difficulty with dressing including putting on shoes and socks? | **0.68** | 0.01 | 0.24 | -0.06 | |  |
| Because of a health problem, do you have any difficulty with walking across a room? | **0.79** | 0.17 | 0.07 | 0.00 | |  |
| Because of a health problem, do you have any difficulty with bathing or showering? | **0.92** | 0.06 | 0.01 | -0.03 | |  |
| Because of a health problem, do you have any difficulty with eating, such as cutting your food? | **0.93** | -0.04 | -0.03 | 0.04 | |  |
| Because of a health problem, do you have any difficulty with getting in or out of bed? | **0.81** | -0.07 | 0.20 | -0.12 | |  |
| Because of a health problem, do you have any difficulty with using the toilet, including getting on and off the toilet or squatting? | **0.84** | -0.02 | 0.11 | -0.08 | |  |
| Because of a health problem, do you have any difficulty with preparing a hot meal? | **0.93** | 0.01 | -0.08 | 0.13 | |  |
| Because of a health problem, do you have any difficulty with shopping for groceries? | **0.84** | 0.08 | 0.01 | 0.09 | |  |
| Because of a health problem, do you have any difficulty with taking medications (if you take any or needed to do so)? | **0.88** | -0.02 | -0.09 | 0.26 | |  |
| Because of a health problem, do you have any difficulty managing your money? | **0.85** | -0.04 | -0.03 | 0.28 | |  |
| How is your vision (using glasses)? Is it... | **0.30** | 0.00 | 0.14 | 0.28 | |  |
| How is you hearing/auditory range (using hearing aid or auditory device)? | 0.18 | 0.05 | 0.17 | **0.31** | |  |
| Copying of figures 2001-2003 | 0.23 | -0.07 | 0.04 | **0.68** | |  |
| Visual scanning | 0.19 | 0.06 | 0.02 | **0.61** | |  |
| Visual memory 2001-2003 | 0.10 | -0.03 | 0.16 | **0.66** | |  |
| Verbal learning | -0.08 | 0.35 | -0.01 | **0.64** | |  |
| Verbal recall | -0.03 | 0.42 | -0.12 | **0.67** | |  |

Notes: χ^2^=4,985.58, df=402, RMSEA=0.034; 90%CI=0.033-0.035, CFI=0.977

| Geomin factor correlations | | | | |
| --- | --- | --- | --- | --- |
|  | Factor 1 | Factor 2 | Factor 3 | Factor 4 |
| Factor 1 | 1 |  |  |  |
| Factor 2 | 0.464* | 1 |  |  |
| Factor 3 | 0.650* | 0.439* | 1 |  |
| Factor 4 | 0.147* | 0.003 | -0.058 | 1 |

*Notes*: * p-value<0.05

**Supplementary Table S8: Model selection criteria-one group analysis**

| Model of Change | CFI | RMSEA | 90% | chi-square | df | BIC | SSABIC |
| --- | --- | --- | --- | --- | --- | --- | --- |
| Linear | 0.995 | 0.032 | 0.026-0.038 | 76.99 | 5 | 331,015.70 | 330,987.09 |
| Quadratic | 0.998 | 0.048 | 0.035-0.062 | 33.04 | 1 | 331,009.98 | 330,968.67 |
| Latent Basis | 0.998 | 0.024 | 0.017-0.033 | 28.07 | 3 | 330,985.89 | 330,950.94 |

*Notes*: CFI: comparative fit index; RMSEA: root mean square of approximation; df: degrees of freedom; BIC: Bayesian information criterion; SSABIC: sample-size adjusted BIC

**Supplementary Table S9: Regression of growth factors (intercept and slope) on the covariates in the conditional 4-class model^†^**

|  | Class 1-Decliners | Class 2-Moderate-stable | Class 3-High-stable | Class 4-Low-stable |
| --- | --- | --- | --- | --- |
|  | (n=3,161) | (n=1,824) | (n=8,247) | (n=756) |
|  | **Estimate (SE)** | | | |
| **Intercept** |  |  |  |  |
| Mean | 68.27 (0.40)** | 56.40 (1.31)** | 75.83 (0.29)** | 39.69 (1.32)** |
| Physical Activity (Non-physically active vs active) | -1.91 (0.61)** | -1.50 (1.54) | -0.38 (0.36) | 0.76 (4.03) |
| Ever smoking (Never smokers vs current/former smokers) | 0.43 (0.70) | -0.56 (1.25) | 0.06 (0.30) | -2.06 (2.03) |
| Drinking of alcohol (Never and no drinkers vs drinkers) | -0.32 (0.54) | -0.89 (1.47) | -0.38 (0.23) | -3.21 (2.35) |
| **Slope** |  |  |  |  |
| Mean | -28.29 (1.31)** | -7.71 (1.36)** | -11.52 (0.31)** | -6.46 (6.94) |
| Physical Activity (Non-physically active vs active) | 2.55 (1.91) | 1.19 (1.54) | -0.11 (0.34) | 20.32 (9.96)** |
| Ever smoking (Never smokers vs current/former smokers) | -2.77 (2.19) | -0.24 (1.67) | -0.25 (0.38) | 11.66 (7.75) |
| Drinking of alcohol (Never and no drinkers vs drinkers) | -2.58 (1.49)* | 1.28 (1.49) | -0.58 (0.29)** | 24.58 (14.09)** |
| **Death Probability** |  |  |  |  |
|  | 0.81 (0.02)** | 0.26 (0.04)** | 0.05 (0.01)** | 0.95 (0.03)** |

*Notes*: n: number of participants; SE: standard errors. **: statistically significant in 0.05 level; *: statistically significant in 0.10 level; **†:** adjusted for sex, age and education level.
